# Successful recovery from COVID-19 pneumonia with awake early self-proning

**DOI:** 10.1186/s42077-021-00184-0

**Published:** 2021-10-15

**Authors:** Fulya Yılmaz, Koray Bas

**Affiliations:** 1 Department of Anesthesiology and Reanimation, University of Health Sciences Izmir Bozyaka Training and Research Hospital, Saim Çıkrıkçı Caddesi, No. 59, İzmir, Karabağlar Turkey; 2 Department of General Surgery, Faculty of Medicine, İzmir Bakırçay University, İzmir, Menemen Turkey

**Keywords:** Prone positioning, Awake patient, Non-intubated patient, Non-invasive mechanical ventilation, COVID-19

## Abstract

**Background:**

Since COVID-19 global pandemic, “early awake proning in non-intubated patients with COVID-19” has been suggested as anecdotal evidence. Hereby, we report an awake and non-intubated patient with COVID-19 pneumonia who was successfully managed with early self-proning.

**Case presentation:**

A 68-year-old male presented to the emergency department with a respiratory distress. He was non-smoker and denied any significant past medical history. His chest computed tomography scan showed “ground glass opacities” and “consolidation areas” located especially in the peripheral sites of both lungs which were consistent with a coronavirus pneumonia and reverse transcription polymerase chain reaction amplification by a nasopharyngeal swab sample for SARS-Cov-2 was also positive. His initial therapy with *hdroxychloroquine* and *favipiravir was* started. Due to deterioration of the patient’s oxygenation, he was transferred to the intensive care unit for further treatment with non-invasive mechanical ventilation on supine position and intermittent “awake early self-proning positioning” was applied. Additionally, antibiotherapy, anticoagulant therapy, and convalescent plasma therapy were also administered to the patient. On the 17th day of the ICU admission, he was transferred back to the ward. And the patient was discharged from the hospital on the 19th day of his initial admission.

**Conclusions:**

Although some case reports and small case series initially noted potential improvement in oxygenation by awake proning, further research is required to evaluate the exact benefits and proper applications of prone positioning in awake patients with COVID-19 pneumonia.

## Background

Prone positioning is a well-established and evidence-based practice for patients with acute respiratory distress syndrome (ARDS) undergoing invasive mechanic ventilation (Prasad & Visrodia, n.d.; Koeckerling et al., n.d.; Elharrar et al., 2020). But there is a limited data that exist for awake and non-intubated patients (Koeckerling et al., n.d.; Ng et al., 2020)). By COVID-19 global pandemic, “early awake proning non-intubated patients with COVID-19” was suggested as anecdotal evidence (Prasad & Visrodia, n.d.)). Hereby, we report an awake, non-intubated patient with COVID-19 pneumonia who was successfully managed with early self-proning.

## Case presentation

**Fig. 1:**
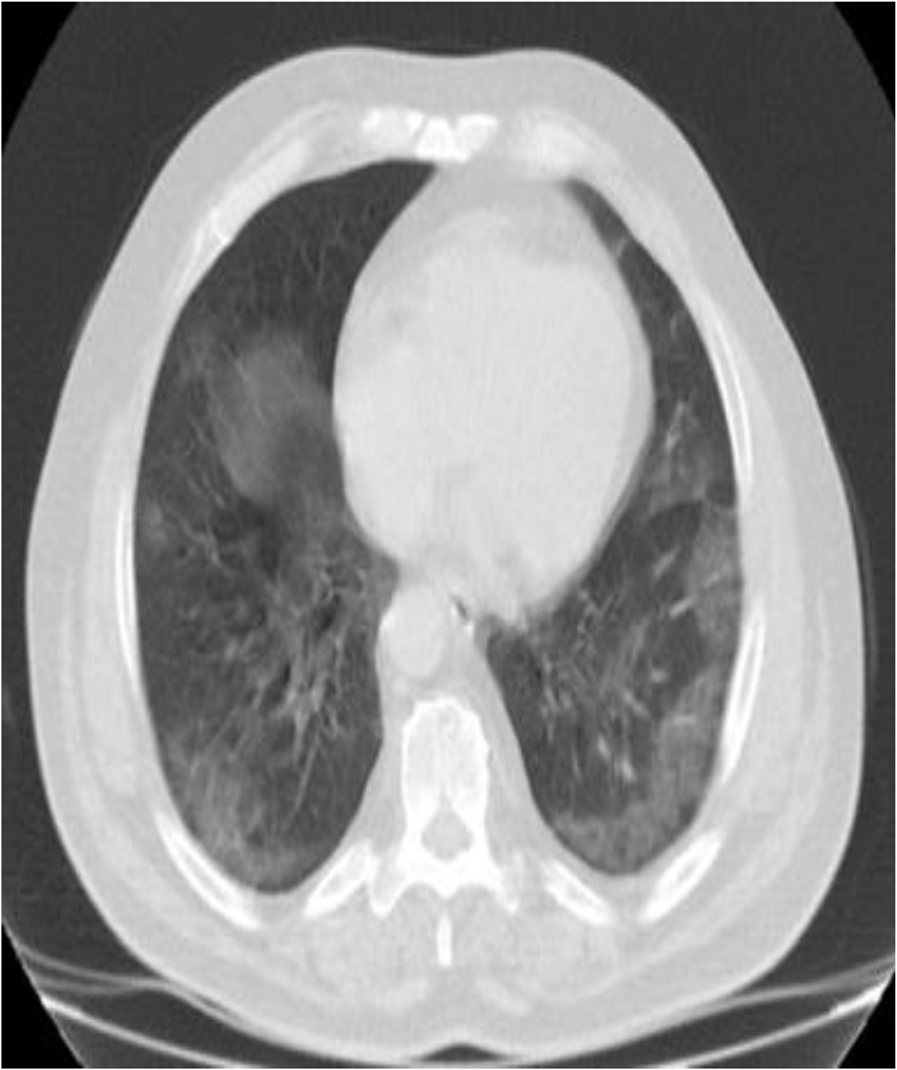
Chest CT findings on admission

The written informed consent from the patient was achieved. A 68-year-old male presented to the emergency department with a respiratory distress. He was non-smoker and denied any significant past medical history. His chest computed tomography (CT) scan showed “ground glass opacities” and “consolidation areas” located especially in the peripheral sites of both lungs which were consistent with coronavirus pneumonia (Fig. 1), and reverse transcription polymerase chain reaction (RT-PCR) amplification by a nasopharyngeal swab sample for SARS-Cov-2 was also positive. His hemodynamic parameters were stable at the admission and the initial physical examination was normal apart from bilateral pulmonary rales and rhonchi by chest auscultation. The laboratory values were also in normal ranges except a lower partial arterial oxygen pressure (PaO2) in the arterial blood gas sample. RT-PCR amplification testing by a nasopharyngeal swab sample for SARS-Cov-2 revealed positive. Then, the patient was transferred to the specialized COVID-19 ward of the hospital and *hydroxychloroquine* (2 × 200 mg/day orally) and *favipiravir* (2 × 600 mg/day orally) were started as an initial treatment. On the 3rd day of the treatment, due to the deterioration of the patient’s oxygenation, the patient was transferred to the intensive care unit (ICU) for further treatment with non-invasive mechanical ventilation (NIMV) on supine position. Antibiotherapy (400 mg oral moxifloxacin once/day), anticoagulant therapy (4000 IU/0.4ml anti-Xa subcutaneous injection once/day), and 3 sessions of convalescent plasma therapy were administered to the patient. In addition to these standard treatments, “awake early self-proning” was started to be performed as soon as he was transferred to the ICU and was applied as long as possible according to his comfort daily. From the moment the patient was taken to the ICU, it was ensured that he remained in the prone position whenever possible. Arterial blood gas samples were taken after being in the prone position for 1 and 2 h, and during the days he stayed in the ICU also showed the positive effects of the prone position (Table 1). We continued to apply “awake early self-proning” that the patient stays in the ICU.

**Table 1:** Intermittent arterial blood gas analysis during ICU stay

Arterial blood gas samples analysis time		pH	PO2	pCO2	HCO3	Lactat
**On admission**	**Supine**	7.50	75.8	31.3	26.3	0.7
	**After an hour awake self-proning**	7.49	88.6	32.5	26.8	1.7
	**After 2-h awake self-proning**	7.44	105	34.8	24.8	1.0
**1st day of admission**	**Supine**	7.46	64	35.9	26	1.7
	**After an hour awake self-proning**	7.43	70.4	38.3	25.9	1.4
	**After 2-h awake self-proning**	7.46	92.6	36.4	26.8	1.4
**2nd day of admission**	**Supine**	7.41	66.5	40.8	25.9	1.0
	**After an hour awake self-proning**	7.46	83.8	36	27	0.8
	**After 2-h awake self-proning**	7.41	91	43	27	1.0
**3rd day of admission**	**Supine**	7.47	68	40	29	1.1
	**After an hour awake self-proning**	7.45	72	41	28	1.3
	**After 2-h awake self-proning**	7.48	110	38	29	1.2
**12th day of admission**	**Supine**	7.49	58	40	30	1.1
	**After an hour awake self-proning**	7.46	78	39	28	0.8
	**After 2-h awake self-proning**	7.38	115	38	22.9	1.4
**During discharge from ICU**		7.39	119	43	25	0.7

**Fig. 2:**
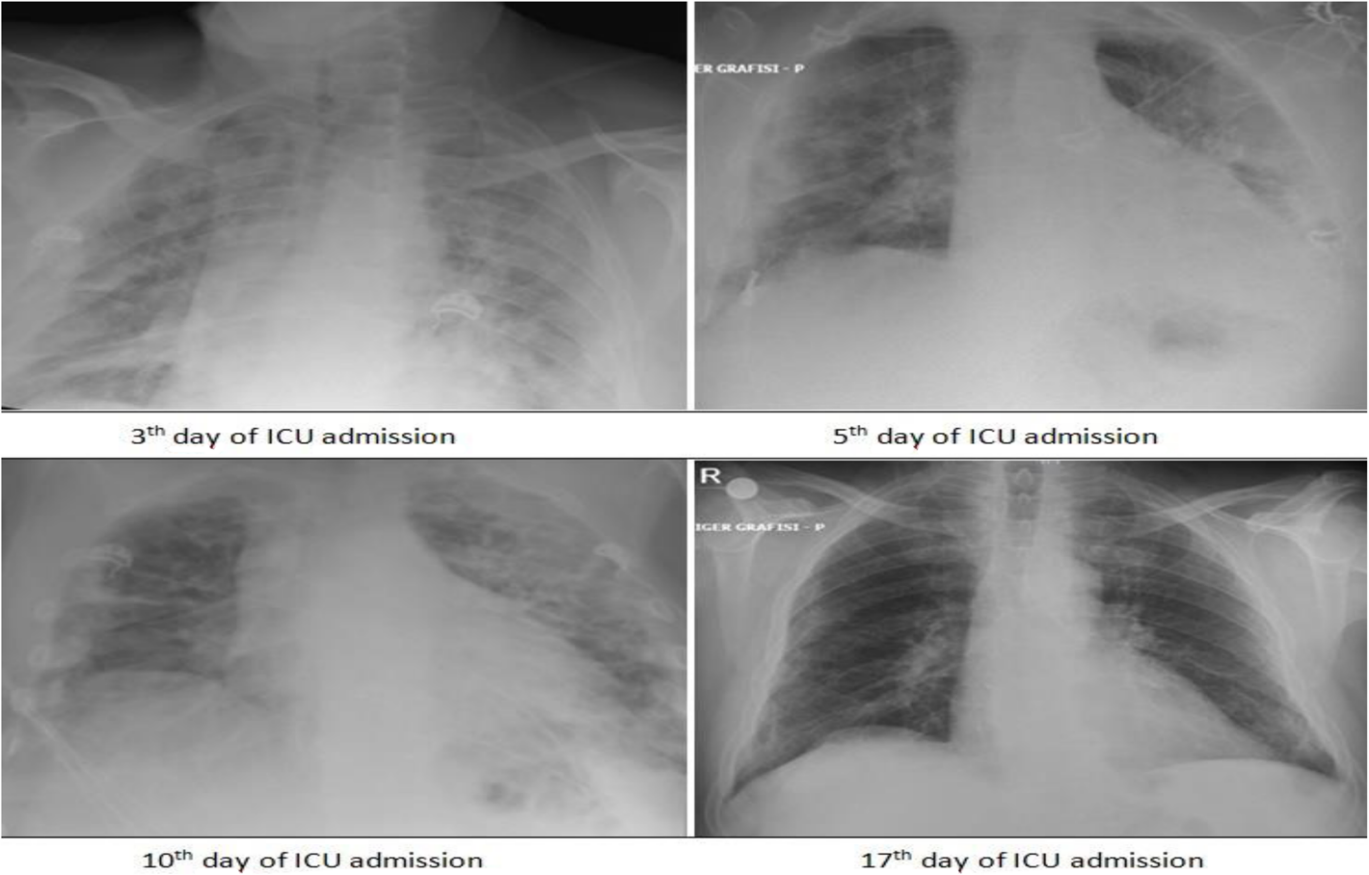
Intermittent PA chest radiographs during ICU stay

It was observed that the patient’s oxygenation improved with the periods of each awake proning. And each improvement was confirmed by both increasing peripheral oxygen saturation and PaO_2_ level in arterial blood gas sample (Table 1). He did not require any sedation in the course of prone positioning. The patient was followed up with intermittent chest X-rays during the ICU stay (Fig. 2).On the 5th and 10th days of the ICU admission, RT-PCR amplification tests by a nasopharyngeal swab sample for SARS-Cov-2 were repeated and both turned out negative. On the 17th day of the ICU admission, after being successfully weaned off supplementary oxygen with resting O_2_ saturation level of 96% and remarkably improved chest radiography, he was transferred back to the ward. Then, the patient was discharged from the hospital on 19th day of his initial admission.

## Discussion

Prone positioning in awake patient is a simple maneuver without needing any additional cost, man power, or special equipment (Caputo et al., 2020). Proning also reduces ventral-dorsal transpulmonary pressure difference, supports alveolar recruitment and improves secretion management (Prasad & Visrodia, n.d.; Ng et al., 2020; Sartini et al., 2020). On the other hand, because of improved lung secretion and increased coughing, following the prone positioning may lead to increase viral contamination risk for the patient’s environment (Koeckerling et al., n.d.). Some general contraindications of prone positioning are spinal instability, facial or pelvic fractures, unstable chest injuries, delirium, and vomiting (Prasad & Visrodia, n.d.).

Thompson et al. (Thompson et al., 2020) reported that prone positioning improves oxygenation in awake, non-intubated patients with hypoxemic respiratory failure due to COVID-19. Similarly, Ng et al. (Ng et al., 2020) evaluated “awake prone positioning” on 10 non-intubated oxygen-dependent COVID-19 pneumonia patients and reported that there were improving oxygenation and reducing need for mechanic ventilation. Caputo et al. (Caputo et al., 2020) demonstrated improved oxygen saturation by “early use of self-proning” in awake non-intubated patients with COVID-19 in the emergency department. It was also evaluated that the feasibility, efficacy, and the tolerance of prone positioning in 24 awake patients with COVID-19 by Elharrar et al. This study also claimed that there was an improvement on oxygenation in patients who can tolerate prone position for more than 3 h (Elharrar et al., 2020). Sartini et al. (Sartini et al., 2020) assessed non-invasive ventilation in addition to proning in 15 COVID-19 patients in general wards, and they reported improved oxygenation.

Results and limitations of all studies were similar in the current literature. They reported improvement in oxygenation with “awake proning.” But, they included only small number of patients without any control groups.

Unlike from the studies above, we applied “early awake self-proning in an ICU patient.” The patient stated that he felt more comfortable with the prone position and preferred to stay in prone as much as possible. Since Xu et al. (Prasad & Visrodia, n.d.) did not describe any exclusion criteria for awake proning, we might claim that it can be applied in the majority of patients with COVID-19 pneumonia. The comfort of the patient looks like one of the most important factors on the duration of awake prone positioning in our experience.

In spite of its promising advantages, there are still some crucial questions to answer about awake proning in COVID-19 patients such as “Which criteria should we use to identify appropriate candidates for awake prone positioning?” “What is the optimal timing and the duration of awake proning?” and “If it fails, how should we monitor proning patients to avoid any delay in intubation?” (Bower & He, 2020).

## Conclusions

Although some case reports and small case series initially noted a potential improvement in oxygenation by awake proning, as we present by this report, further research is required to evaluate the exact benefits and proper applications of prone positioning in awake patients with COVID-19 pneumonia.

## Data Availability

The datasets used and/or analyzed during the current study are available from the corresponding author on reasonable request.

## Abbreviations

CT: Computed tomography
RT-PCR: Reverse transcription polymerase chain reaction
PaO2: Partial arterial oxygen pressure
ICU: Intensive care unit
NIMV: Non-invasive mechanical ventilation
